# A defense-offense multi-layered regulatory switch in a pathogenic bacterium

**DOI:** 10.1093/nar/gkv001

**Published:** 2015-01-27

**Authors:** Mor Nitzan, Pierre Fechter, Asaf Peer, Yael Altuvia, Delphine Bronesky, François Vandenesch, Pascale Romby, Ofer Biham, Hanah Margalit

**Affiliations:** 1 Racah Institute of Physics, The Hebrew University, Jerusalem 91904, Israel; 2 Department of Microbiology and Molecular Genetics, IMRIC, Faculty of Medicine, The Hebrew University, Jerusalem 91120, Israel; 3 Architecture et Réactivité de l'ARN, Université de Strasbourg, CNRS, IBMC, Strasbourg F-67084, France; 4 CIRI, International Center for Infectiology Research,Lyon, France; 5 Inserm, U1111, Lyon, France; 6 École Normale Supérieure de Lyon, Lyon, France; 7 Université Lyon 1, Lyon, France; 8 CNRS, UMR5308, Lyon, France

## Abstract

Cells adapt to environmental changes by efficiently adjusting gene expression programs. *Staphylococcus aureus*, an opportunistic pathogenic bacterium, switches between defensive and offensive modes in response to quorum sensing signal. We identified and studied the structural characteristics and dynamic properties of the core regulatory circuit governing this switch by deterministic and stochastic computational methods, as well as experimentally. This module, termed here Double Selector Switch (DSS), comprises the RNA regulator RNAIII and the transcription factor Rot, defining a double-layered switch involving both transcriptional and post-transcriptional regulations. It coordinates the inverse expression of two sets of target genes, immuno-modulators and exotoxins, expressed during the defensive and offensive modes, respectively. Our computational and experimental analyses show that the DSS guarantees fine-tuned coordination of the inverse expression of its two gene sets, tight regulation, and filtering of noisy signals. We also identified variants of this circuit in other bacterial systems, suggesting it is used as a molecular switch in various cellular contexts and offering its use as a template for an effective switching device in synthetic biology studies.

## INTRODUCTION

**Figure 1.**
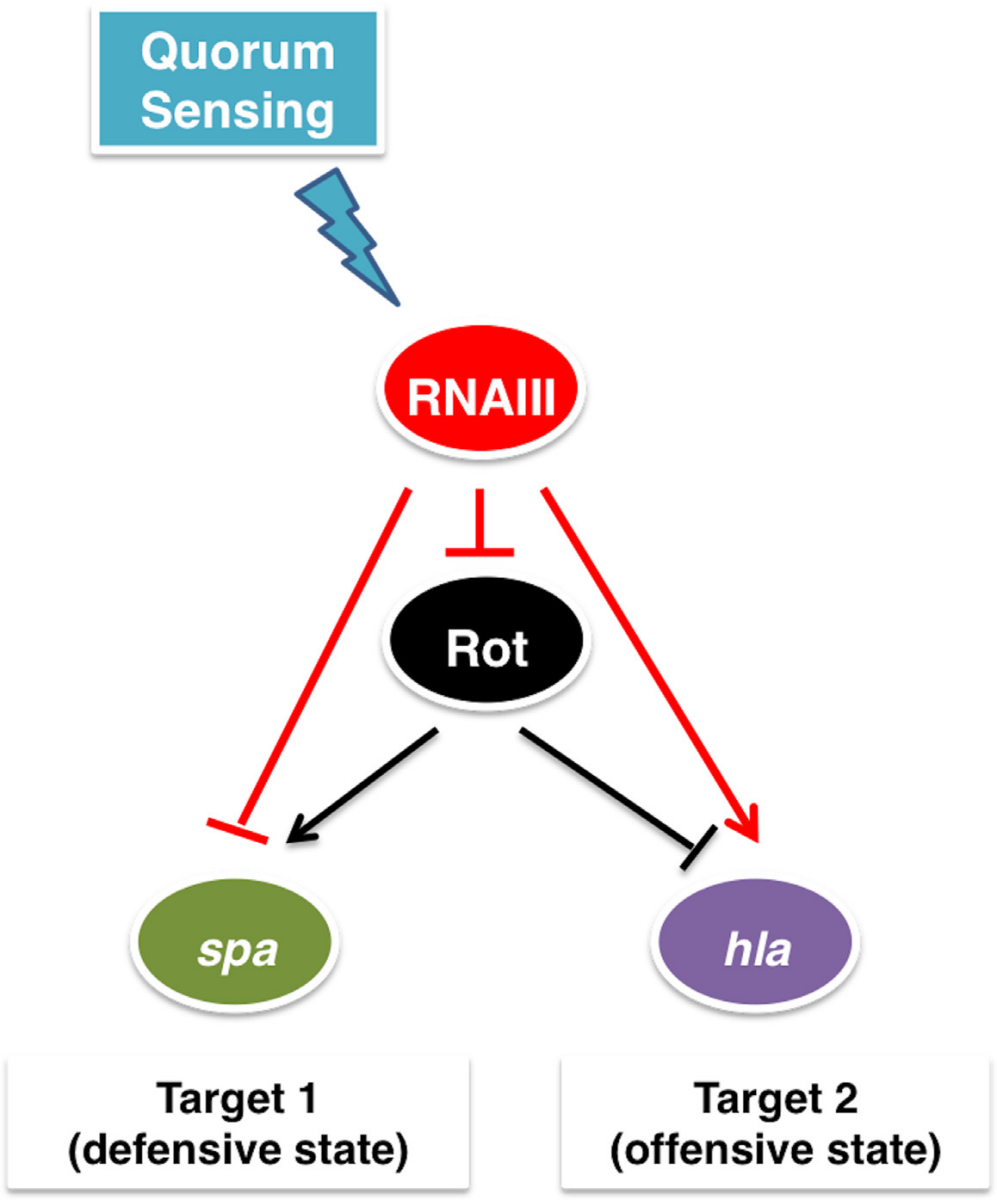
The *Staphylococcus aureus* Double Selector Switch (DSS). Arrows indicate positive regulation and T-shaped arrows indicate negative regulation (red for regulation by a sRNA and black for regulation by a TF). Target 1 genes encode adhesins and defensive proteins, such as the gene *spa* encoding protein A, while target 2 gene, *hla*, encodes α-hemolysin (Hla).

Bacteria may undergo major transitions during their cellular life, such as transitions between aerobic and anaerobic metabolism, between motile and sessile lifestyles or between colonization and dissemination (or spreading) in case of bacterial pathogens. Adaptation to a new environment usually requires changes in gene expression programs that need to be precisely controlled, turning on the expression of genes required for the new condition and switching off the expression of unnecessary genes (e.g. (1–8)). Since many transcription factors (TFs) in bacteria act as activators of gene expression for some genes and repressors for others, such a switch can be potentially achieved at the transcription regulation level (2,9–12). However, since bacterial regulatory small RNAs (sRNAs) are capable of both repressing and activating genes they also can control such switching (13–19). Indeed we recognized a sophisticated switching module comprising a transcription factor (Rot) and a small RNA (RNAIII) in *Staphylococcus aureus*, an opportunist Gram-positive pathogenic bacterium that switches between defensive and offensive modes in response to quorum sensing signal (Figure 1) (20–22). Quorum sensing in bacteria, or cell-density sensing, is a process that involves communication through secreted signaling molecules (23). In the defensive mode the bacteria express cell surface proteins, which confer *S. aureus* the ability to adhere to cells and tissue matrix and form biofilm, and proteins that enable the bacterium to evade the host immune system (referred to as defensive genes). In the offensive mode the bacteria secrete toxins such as superantigens that stimulate the immune system, exfoliative toxins promoting intra-dermal cleavage, and pore-forming toxins that form tunnels and pores in the membranes of the host cells (referred to as offensive genes) (22). The expression of these accessory factors is highly coordinated and is closely linked to the metabolism and biological requirements of *S. aureus* (24). Switching between the defensive and offensive modes has been considered a result of a complex network of regulatory interactions (25). Within this network, we isolated and analyzed in detail the properties of the minimal structure that produces the phenotypic switching. The two-layered switch we identified includes two regulators (Figure 1): (i) the transcription factor Rot, which is active when cell density is low, and simultaneously activates adhesins and defensive genes and represses the offensive genes (26–28); (ii) the regulatory RNA, RNAIII, which is activated when cell density increases, and simultaneously represses post-transcriptionally both *rot* (28) and the defensive genes while activating the translation of the exotoxin *hla* (29).

The *agr* system, which senses the local population density and regulates the temporal expression of many virulence factors, has been recognized as one of the pivotal global regulators of *S. aureus* pathogenesis and physiology (7,25,30). It is composed of two divergent transcripts: RNAII, encoding a quorum sensing cassette and a two-component system, and RNAIII, the regulatory small RNA that is the main effector of the system. The quorum sensing cassette produces and secretes the autoinducer peptide (AIP), which upon a threshold concentration activates the transcription of the entire *agr* system and of RNAIII. RNAIII acts as a negative regulator of *rot* and the defensive genes through base-pairing with their mRNAs, inhibiting their translation initiation (27,28). The endoribonuclease III (RNase III) is then recruited to cleave the sRNA–mRNA complexes (28,31–32). RNAIII also base-pairs with the mRNA of the offensive gene *hla* and enhances its translation by releasing an otherwise blocked ribosome binding site (29). The dynamics of the circuit we identified has not been studied yet. Furthermore, this circuit comprises regulatory structures that have not yet been analyzed as an integrated regulatory unit. From a horizontal point of view, it forms a structure of two integrated single-layered switches, one governed by the TF and one governed by the sRNA. From a vertical point of view, it is built of two combined double-layered coherent feed-forward loops (FFLs), involving both transcriptional and post-transcriptional regulators. We termed this circuit hereinafter Double Selector Switch (DSS) (‘Double’, for its two layers of regulation, and ‘Selector Switch’, for the switch between two alternative gene expression programs). In this study, we explore by deterministic and stochastic methods the role of transcriptional and post-transcriptional regulations in this module. We study its unique properties, including fine-tuned coordination of target gene expression, filtering of transient signals, and prevention of expression leakage. We present experimental data that supports the theoretical model dynamics and the specific coordination of target expression. In addition, we explore variants of this circuit and their role in phenotypic switching in other bacterial systems.

## MATERIALS AND METHODS

### Deterministic model

The wiring diagram of the DSS (Figure 1) was converted into a set of coupled ordinary differential equations (ODEs) under the assumption of mass action kinetics for all reactions. The model describes the temporal variation in the levels of all relevant molecular types, where *N*_*X*_ is the number of molecules of type *X* in the cell, modeled as a continuous quantity. Note that for simplicity we used the same parameter values for the various molecules (e.g. the mRNAs of the TF and of target genes 1 and 2 are generated at the same rate; the mRNAs of the TF and target genes 1 and 2 bind to the sRNA at the same rate). In order to capture the generic behavior of the DSS, the functional forms of the model were chosen as the simplest possible forms that are consistent with the established knowledge of the types of transcriptional and post-transcriptional interactions within the system, as described in the ‘Introduction’ section (similarly to, e.g. (33)). This implies, for example, that for the post-transcriptional regulation we assume stoichiometric behavior with no dissociation of the sRNA–mRNA complex. This was also demonstrated experimentally, since the various RNAIII–mRNA targets were found to be highly stable and the translationally repressed mRNAs were rapidly degraded in a manner dependent on the endoribonuclease III (28,31). The rate equations describe the following processes: the sRNA (*N*_*S*_) is generated at rate *g*_*S*_ and degraded at rate *d*_*S*_. In addition, it binds separately and irreversibly to the mRNA transcripts of the TF (*N*_*mT*_), target 1 (*N*_*m*1_) and target 2 (*N*_*m*2_) at rate *b*_*S*_. The TF mRNA is generated at rate *g*_*m*_ and degraded at rate *d*_*m*_, while the TF protein (*N*_*PT*_) is synthesized from mRNA at rate *g*_*P*_ and degraded at rate *d*_*P*_. The TF protein binds to gene 1 and gene 2 promoters to form TF-promoter complexes (*N*_*T*1_ and *N*_*T*2_, respectively) at rate *b*_*T*_, and unbinds at rate *u*_*T*_. Note that we assume no cooperative binding of the TF, and that no more than one TF protein can be bound to a certain promoter at a given time, and thus, the model ensures that 0 ≤ *N*_*T*1_, *N*_*T*2_ ≤ 1. The TF up-regulates the transcription of gene 1, leading to an overall transcription rate *g*_*m*_*N*_*T*1_, while it down-regulates the transcription of gene 2, leading to an overall transcription rate *g*_*m*_(1 − *N*_*T*2_). The mRNAs of gene 1 and gene 2 are degraded at rate *d*_*m*_. Gene 1 proteins (*N*_*P*1_) are translated from mRNAs at rate *g*_*P*_ and are degraded at rate *d*_*P*_. The three complexes sRNA-gene 1 mRNA, sRNA-gene 2 mRNA and sRNA-TF mRNA (*N*_*S*1_, *N*_*S*2_ and *N*_*ST*_, respectively) are degraded at rate *d*_*Sm*_. While the sRNA down-regulates gene 1, it up-regulates gene 2, enabling its mRNA translation. Therefore, gene 2 proteins (*N*_*P*2_) are translated from sRNA-bound mRNAs at rate *g*_*P*_ and are degraded at rate *d*_*P*_. Biologically, a low rate of free mRNA translation may exist for target 2, in which case a second-order translation term would be added to the equation of target 2 protein. The variables used in this model are listed in Table 1 and the parameter values used for the simulations are reported in Supplementary Table S1. The rate equations describing the DSS take the form:

(1a) \begin{equation*} \frac{d N_S}{dt} = g_s - b_S N_S (N_{mT} + N_{m1} + N_{m2}) - d_S N_S \end{equation*}

(1b) \begin{equation*} \frac{d N_{mT}}{dt} = g_m - b_S N_S N_{mT} - d_m N_{mT} \end{equation*}

(1c) \begin{equation*} \frac{d N_{ST}}{dt} = b_S N_S N_{mT} - d_{Sm} N_{ST} \end{equation*}

(1d) \begin{eqnarray*}\frac{d N_{PT}}{dt}&=&g_P N_{mT}{-}d_P N_{PT}{-}[b_T N_{PT} (1-N_{T1}){-}u_T N_{T1}]- \nonumber \\ && {[}b_T N_{PT} (1-N_{T2}) - u_T N_{T2}] \end{eqnarray*}

(1e) \begin{equation*} \frac{d N_{Ti}}{dt} = b_T N_{PT} (1-N_{Ti}) - u_T N_{Ti}, i=1,2 \end{equation*}

(1f) \begin{equation*} \frac{d N_{m1}}{dt} = g_m N_{T1} - b_S N_S N_{m1} - d_m N_{m1} \end{equation*}

(1g) \begin{equation*} \frac{d N_{m2}}{dt} = g_m (1-N_{T2}) - b_S N_S N_{m2} - d_m N_{m2} \end{equation*}

(1h) \begin{equation*} \frac{d N_{Si}}{dt} = b_S N_S N_{mi} - d_{Sm} N_{Si}, i=1,2 \end{equation*}

(1i) \begin{equation*} \frac{d N_{P1}}{dt} = g_P N_{m1} - d_P N_{P1} \end{equation*}

(1j) \begin{equation*} \frac{d N_{P2}}{dt} = g_P N_{S2} - d_P N_{P2}. \end{equation*}

**Table 1.** Variables of the mathematical models, where *N*_X_ is the number of molecules of type *X* in the cell

Variable	Symbol
sRNA	*N*_*S*_
TF mRNA	*N*_*mT*_
TF protein	*N*_*PT*_
TF protein bound to target promoter	*N*_*Ti*_, *i* = 1, 2
sRNA–mRNA complex	*N*_*Si*_, *i* = 1, 2, *T*
Target mRNA	*N*_*m*__*i*_, *i* = 1, 2
Target protein	*N*_*Pi*_, *i* = 1, 2

The steady state solutions for the number of sRNA molecules, *N*_*S*_, and the number of TF proteins, *N*_*PT*_, are given by

(2a) \begin{eqnarray*} \begin{array}{*{20}l} {N_S = \frac{1}{{2b_S d_S }}\left\{ {b_S (g_S - 2g_m ) - d_S d_m + } \right.} \\ {\left. {\sqrt {[b_S (g_S - 2g_m ) - d_S d_m ]^2 + 4b_S g_S d_S d_m } } \right\}} \\ \end{array} \end{eqnarray*}

(2b) \begin{equation*} N_{PT} = \frac{g_P}{d_P} \frac{g_m}{d_m + b_S N_S}. \end{equation*}

The ODEs were implemented in matlab (MathWorks) and integrated using its built-in solver ode45. The initial conditions were set as the steady state values of the variables.

#### Target coordination

According to the DSS model (Eqation (1)), the equation describing the variation in time of the total (functional) target mRNA level is:

(3) \begin{eqnarray*} &&\frac{d (N_{m1}+N_{S2})}{dt} = \nonumber \\ &&g_m N_{T1} - b_S N_S (N_{m1} - N_{m2}) - d_m N_{m1} - d_{Sm} N_{S2}. \end{eqnarray*}

**Figure 2.**
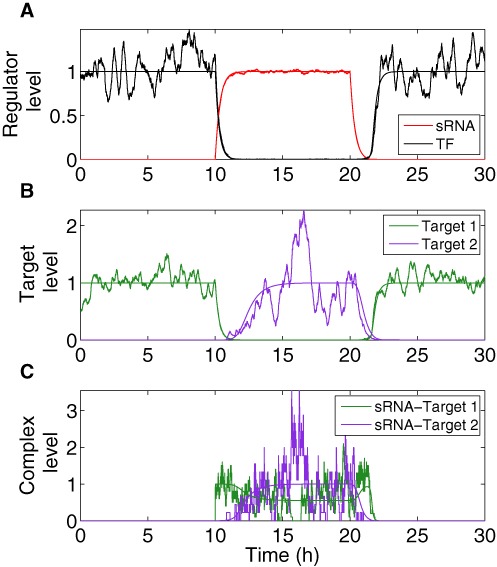
Dynamics of DSS components obtained from deterministic and stochastic analyses. Smooth trajectories that are the result of simulations based on the deterministic model are accompanied by corresponding noisy trajectories that are the result of simulations based on stochastic analysis. The transcription of the top regulator is activated by an external signal at time *t* = 10 h and deactivated by an external signal at time *t* = 20 h. In the Y-axis, the levels are normalized by their respective maximal and minimal deterministic values. (**A**) Expression level of the sRNA (red) and the TF protein (black). (**B**) Target protein level (green: target 1; purple: target 2). (**C**) Level of sRNA-mRNA complexes (green: sRNA complex with target 1 mRNA; purple: sRNA complex with target 2 mRNA). The simulation starts at the OFF state, when the level of the sRNA regulator is 0 and the TF is expressed and active (A), leading to a high level of protein 1 and a low level of protein 2 (B). Upon change in condition (ON step, at *t* = 10 h) the sRNA is activated, leading to a decrease in the level of the TF (A) as well as protein 1 (B), followed by an increase in the level of protein 2 (B). At this stage complexes are formed first between the mRNAs of gene 1 and the sRNA, and later between the mRNAs of gene 2 and the sRNA (C). At the transition to OFF step (at *t* = 20 h) the sRNA level decreases (A), as well as the level of its complexes (C). At this stage the TF level increases (A), leading to a decrease in the level of protein 2, followed by an increase in the level of protein 1 (B). Overall, there is a delay between the down-regulation of target 1 and the up-regulation of target 2 upon ON step and vice versa upon OFF step. The peak in sRNA- target 1 mRNA complexes upon the transition to OFF step occurs due to two opposing processes; the initial decrease in sRNA level and the subsequent increase in target 1 mRNA level. In the stochastic simulations, noise in sRNA level stems from transcription and degradation processes. Noise in protein level (TF and targets) arises due to transcription, translation, degradation and during ON step, due to sRNA level variation as well. Noise in complex levels is due to the combined noises in sRNA and mRNA levels. The parameter values used in these simulations are reported in Table S1.

The RHS of Equation (3) involves variables related to the two regulators: *N*_*T*1_, the promoter-bound TF and *N*_*S*_, the sRNA. Therefore, the total (functional) target mRNA level depends on both the sRNA and the TF levels, and thus changes along with the ON and OFF steps. Since the total target protein level,

(4) \begin{equation*} \frac{d (N_{P1}+N_{P2})}{dt} = g_p (N_{m1}+N_{S2}) - d_P (N_{P1}+N_{P2}). \end{equation*}

depends on the total (functional) target mRNA level, it also changes along with the ON and OFF steps, as observed in Figure 2. This is opposed to the results for two simpler switches: a Simple TF Switch (a DSS structure missing its sRNA–targets interactions) and a Simple sRNA Switch (a DSS structure missing its TF regulator). As is shown in the Supplementary Material, the two targets in the two Simple Switches are inherently symmetrical in their individual incline and decline upon changes in external signal. However, the two targets in the DSS are in general not symmetric, leading to different behaviors and response times upon ON and OFF steps. These results, for the DSS and Simple Switches, were obtained analytically, and are therefore valid for any set of parameter values.

#### Leakage

The leakage level of a target is defined as the ratio between the steady state level under conditions in which the target is repressed and maximal possible level (achieved with no negative regulation) of its protein, $L_X=\frac{N_X}{{N_X}^{max}}$. The leakage of target gene 1 (when the top sRNA regulator is activated) is given by:

(5) \begin{equation*} L_1 = \frac{N_{P1}}{{N_{P1}}^{max}}{=}\frac{N_{P1}}{ \frac{g_m}{d_m} \frac{g_P}{d_P}}{=}\left( \frac{ \frac{b_T}{u_T} N_{PT}}{1+\frac{b_T}{u_T} N_{PT}}\right) \left(\frac{1}{1+\frac{b_S}{d_m}N_S}\right). \end{equation*}

The leakage level of target 1 in a DSS is a product of the leakage level in case the target is only regulated by the TF, such as in the case of a Simple TF Switch (first term on the R.H.S of Equation (5), Supplementary Material Equation S5) and the leakage level in case the target is only regulated by the sRNA, such as in the case of a Simple sRNA Switch (second term on the R.H.S of Equation (5), Supplementary Material Equation S7). Therefore, since each term is <1, the product of both terms is smaller than each individual term, and thus leakage is reduced by the combination of the two layers of regulation. Similarly, the leakage of target gene 2, when the top sRNA regulator is not activated, is given by

(6) \begin{eqnarray*} &&L_2 = \nonumber \\ &&\frac{N_{P2}}{{N_{P2}}^{max}}{=}\frac{N_{P2}}{ \frac{g_m}{d_m} \frac{g_P}{d_P}}{=}\left(\frac{1}{1+\frac{b_T}{u_T} N_{PT}} \right)\left(\frac{b_S N_S}{ d_{Sm} (1+\frac{b_S}{d_m}N_S )}\right). \end{eqnarray*}

Similarly to Equation (5), the leakage level of target 2 is a product of the leakage level in case the target is only regulated by the TF (first term on the R.H.S of Equation (6)) and the leakage level in case the target is only regulated by the sRNA (second term on the R.H.S of Equation (6)). These are again analogous to the two Simple Switches, as described in the Supplementary Material (Equations S6 and S8). To summarize, both targets exhibit reduced leakage due to the combination of transcriptional and post-transcriptional regulations, compared to a single layer of regulation.

### Stochastic model

Analogously to the deterministic analysis, the state of the DSS can be described by the state vector (*N*_*S*_, *N*_*mT*_, *N*_*ST*_, *N*_*PT*_, *N*_*T*1_, *N*_*T*2_, *N*_*m*1_, *N*_*m*2_, *N*_*S*1_, *N*_*S*2_, *N*_*P*1_, *N*_*P*2_). The master equation corresponding to the mathematical model of the DSS, presented above (Equation (1)), describing the time dependence of the probability distribution *P*(*N*_*S*_, *N*_*mT*_, *N*_*ST*_, *N*_*PT*_, *N*_*T*1_, *N*_*T*2_, *N*_*m*1_, *N*_*m*2_, *N*_*S*1_, *N*_*S*2_, *N*_*P*1_, *N*_*P*2_), takes the form:

**Figure:**
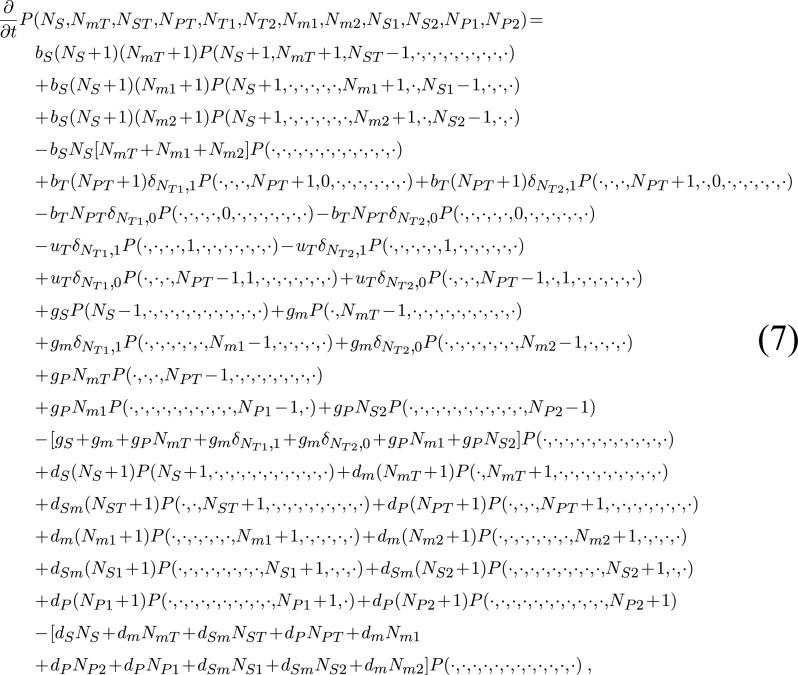


where, for convenience, if there is no change in the state of the variables within the distribution, they are marked by a dot (‘·’).

We used the Gillespie algorithm (34), a kinetic Monte Carlo approach, to generate ‘paths’ of the stochastic process. In this approach it is assumed, for simplicity, that all the stochastic processes at the molecular level are Poisson processes. At each time step the next move is drawn from all possible processes that may take place at that point, where each step is endowed with a suitable weight. After each move the elapsed time is properly advanced, the list of available processes is updated and their new rates are evaluated. The description of stochasticity in biological regulatory processes was previously presented in a similar manner (e.g. (33)).

### Preparation of biological samples

Several bacterial strains were used in this study (Table S2), RN6390, which is a SigmaB-deficient strain (35), and the isogenic mutants, carrying either a deletion of the *rnaIII* gene (nts 1015–1579) (36), or a deletion of the *rot* gene (28). The strains were grown in tryptic soy broth (TSB) medium. RN6390 strain is a strong producer of RNAIII. The transcription profile of this strain is strongly governed by RNAIII, which results in a strong induction of the exoproteins at the stationary phase of growth (37). A pre-culture of 5 ml inoculated with one fresh colony was done overnight and 5 ml were inoculated into 500 ml of fresh TSB and grown with vigorous aeration at 220 rpm at 37°C. Aliquotes were taken every hour for the preparation of total RNA and protein extracts.

### Northern analysis

Total RNAs were prepared using the fast RNA pro blue (MP Biochemicals). Electrophoresis of total RNAs (15 μg) was done on a 1% agarose gel containing 20 mM guanidine thiocyanate and vacuum transfered to nylon membrane. Hybridizations with specific digoxigenin-labeled RNA probes complementary to RNAIII, *rot, spa* or *hla* mRNAs and luminescent detection were carried out as described previously (28). For all experiments, we verified the quantity of 5S rRNA using a digoxigenin-labeled oligonucleotide.

### Western analysis

Strains were grown to post-exponential phase by inoculating 500 ml of TSB medium with an overnight culture (1:100) at 37°C for 7 h. Aliquotes were taken every hour. After centrifugation, protein extracts were obtained by resuspension of the cell pellets in 100 μl of Laemmly buffer (63 mM Tris–HCl pH 6.8, 10% glycerol, 2% sodium dodecylsulphate (SDS), 1 mM β-mercaptoethanol) per OD of culture. Equal amounts of total cellular proteins were separated on 12 or 15% polyacrylamide-SDS gels after boiling the samples 5 min at 95°C in a buffer containing SDS. The gels were then transferred onto polyvinyl difluoride (PVDF) membranes. The membranes were blocked overnight with skimmed milk in TBS and were incubated at 20°C with an appropriate dilution (1:1000 or 1:20000) of a polyclonal antibody to detect α-hemolysin (Abcam 15948) or Rot (generous gift from Dr Frees, Faculty of Life Sciences, Copenhagen, Denmark) for 2 h, followed by another 1 h incubation with a 1:10 000 dilution of anti-rabbit antibody horseradish peroxidase (HRP) conjugated (Sigma). Immunoreactive bands were detected with an Enhanced Chemiluminescence (ECL) detection kit (Pierce). Protein A was detected either by incubation with the anti-rabbit secondary antibody, or by a first incubation of 2 h with a biotin anti-Protein A antibody, followed by 1 h incubation with 0.5 mg/ml HRP-streptavidin. Prestained protein standards (Fermentas) were used for molecular mass estimations. The proteins were detected by autoradiography. The gels were stained by Coomassie blue to verify that the quantity of proteins was homogenous in each sample. All experiments were repeated at least three times with different samples. For each experiment, the bands corresponding to protein A and Hla have been quantified using the software SAFA (38).

## RESULTS

### Dynamical properties of the DSS

To study the dynamical properties of the DSS (Figure 1), we used both computational and experimental methods. We employed mathematical modeling and simulation using both deterministic and stochastic approaches, as well as northern and western analyses (‘Materials and Methods’ section), to follow the variation in the levels of the four components of the circuit as cell density changes (the regulatory RNA RNAIII, the transcription factor Rot, the defensive gene *spa* and the offensive gene *hla*). First, we described the DSS by a set of coupled ODEs followed by simulations to deterministically study the temporal variation in the levels of all molecular types involved. In order to account for stochastic effects and be able to measure the distribution of possible levels of the DSS components, we constructed the master equation describing the temporal variation of probability of the DSS to be in different states in terms of its variables. We study the stochastic behavior of the DSS by generating trajectories, representing exact samples from the probability distribution that is the solution of the master equation, using the Gillespie algorithm (34). For generality of the conclusions from the computational analysis we refer to high and low cell density as the ON and OFF states of the circuit, respectively, and to the components of the circuit as the top regulator sRNA (RNAIII in the case of *S. aureus*), the bottom regulator TF (Rot), target gene 1 (*spa*, encoding Protein A) and target gene 2 (*hla*, encoding α-hemolysin (Hla)).

#### Coordination of target gene expression

**Figure 3.**
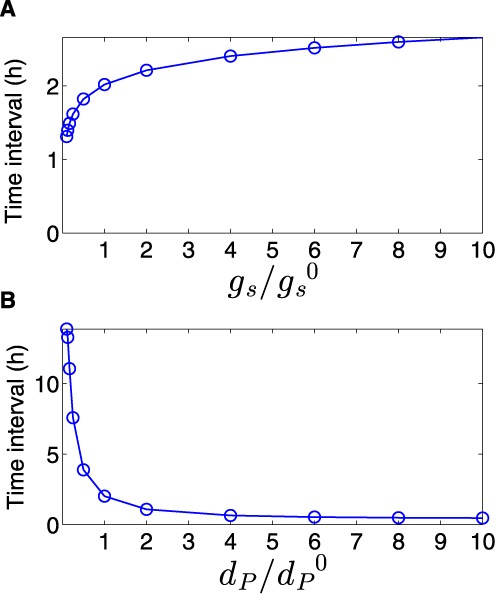
Time interval between the down-regulation of target 1 and up-regulation of target 2. The time interval in this analysis is defined as the difference between the time it takes for targets 1 and 2 to reach halfway to their new steady states upon activation of the sRNA. The time interval, obtained from simulations based on the deterministic model, increases with sRNA transcription rate (**A**) and decreases with TF degradation rate (**B**). The values of $g_s^0$ and $d_p^0$ are reported in Table S1.

**Figure 4.**
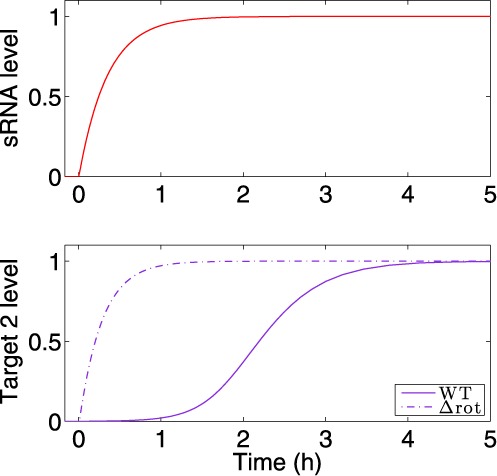
The delay in target 2 expression is abolished in a circuit lacking two layers of regulation. The expression pattern of target 2 under regulation of the DSS was compared to its expression dynamics under the regulation of a Simple sRNA Switch, governed by the sRNA and lacking the TF, using simulations based on the deterministic model. The transcription of the sRNA is activated by an external signal at time *t* = 0 h. Top panel: Expression level of the sRNA (red). Bottom panel: Target 2 protein level. Upon ON step, target 2 rises more quickly for the Simple sRNA Switch (dashed line) than for the DSS (solid line). In the Y-axis, the levels are normalized by their respective maximal and minimal values. The parameter values used in these simulations are reported in Table S1.

**Figure 5.**
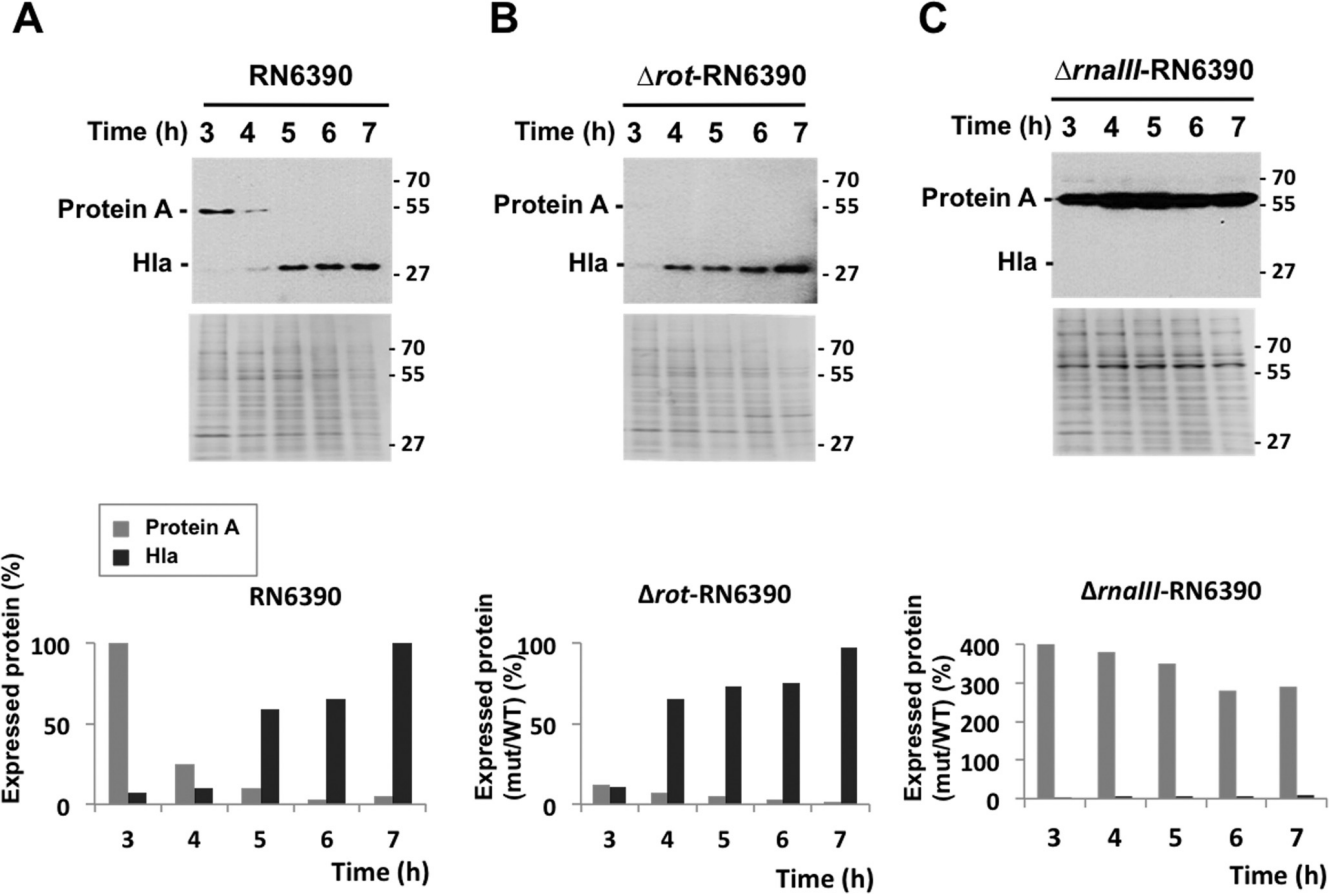
Dynamics of DSS components determined experimentally. Western blots showing the synthesis of Protein A and Hla in the wild-type RN6390 (**A**), the mutant Δrot-RNA6390 (**B**) and ΔRNAIII-RN6390 (**C**) strains. The two proteins were revealed from total cell extracts by immunoblotting (‘Materials and Methods’ section). The quantity of the total proteins was controlled and adjusted in each lane. As the internal loading control, the total proteins were labeled using coomassie blue staining of the same samples used for the immunoblotting. The proteins were extracted hourly at different times of growth. Molecular weight markers were run in parallel. For each experiment, the bands corresponding to protein A and α-hemolysin have been quantified using the software SAFA (38). Several proteins from the loading controls, which do not vary during growth, were used to normalize the results. All data are given as a ratio of the protein yield divided by the highest level observed for each protein in RN6390 (at 3 h for Protein A and at 7 h for α-hemolysin). The experiments were carried out at least three times from different samples with high reproducibility.

The most prominent feature of the circuit is the special coordination of target gene expression (Figure 2). In Figure 2 A, we show the deterministic and stochastic results for the dynamical change in the regulators. Upon ON step (high cell density) the level of the top regulator RNA increases, leading to down-regulation of the bottom regulator TF, whose level decreases. Simultaneously, the level of target 1 decreases followed by an increase in the level of target 2 (Figure 2 B). An opposite transition can be observed upon OFF step (low cell density). It is remarkable that target 2 level starts to increase only after target 1 level has decreased upon ON step, and vice versa upon OFF step, leading to a short overlap time in the expression of the two targets (see ‘Discussion’ section). This is opposed to the simultaneous switching that occurs in Simple Switches (Supplementary Material). Notably, we repeated the simulations for a wide range of parameter values, covering a range between 0.5× and 2× of the typical values reported in Table S1 and obtained qualitatively similar dynamics. This was done in order to clarify that the dynamic behavior observed in Figure 2 is the generic behavior of the system within an extended parameter range and does not require fine-tuning of parameters. We also tested how the time interval between the down-regulation of target 1 and up-regulation of target 2 upon ON step is affected by the variations in two parameters: the generation rate of the sRNA and the degradation rate of the transcription factor protein (Figure 3 and ‘Discussion’ section). Upon ON step, the decrease in target 1 level is promoted by the increase in sRNA level, while the increase in target 2 level is bottlenecked by the decrease of its TF repressor. This means that while the TF is post-transcriptionally down-regulated by the sRNA, there are existing TF proteins that need to be degraded in order to fully relieve the inhibition of target 2 translation. In addition, the steady state level of target 2 during ON step increases for higher sRNA generation rate. Therefore altogether, the time interval between the changes in the targets’ levels upon ON step increases with sRNA generation rate and decreases with TF protein degradation rate (Figure 3). To substantiate the special coordination in gene expression obtained for the DSS we modeled and simulated simple switches derived from the DSS that either lack the transcription regulator or the regulation of the two targets by the sRNA. For both simple switches we obtained a symmetric change in gene expression. Intriguingly, for a simple sRNA switch (a DSS structure lacking its TF regulator), the model predicts that upon ON step target 2 increases substantially faster than in a DSS, thus eliminating the delay between the response of target 1 and target 2, which is a special property of the DSS (Figures 4 and 5 and ‘Discussion’ section).

The experiments, performed for gradual increase in cell density (Figure 5), strongly supported the dynamic pattern revealed by the computational analyses. We followed by northern analysis the steady-state expression levels of RNAIII, *rot* mRNA and mRNAs of their targets, *spa* (corresponding to target 1) and *hla* (corresponding to target 2). These experiments were carried out in the low Sigma B-producing wild-type strain RN6390 expressing RNAIII, as well as in ΔrnaIII–RN6390 and Δrot–RN6390 strains in which the gene encoding RNAIII and rot were deleted, respectively (35) (Supplementary Figure S1). In parallel, the respective protein levels were monitored by western blot analysis (Figures 5, Supplementary Figure S2). The data illustrated the change in RNAIII levels during cell growth, showing accumulation at the late-exponential phase (Supplementary Figure S1). We were unable to detect *spa* mRNA in RN6390, consistent with previous studies (37,39–40). Nevertheless, the synthesis of protein A, which was observed at the beginning of the exponential phase of growth, was strongly reduced after 3 h of culture and was no more detected after 5 h of culture (Figure 5A). Consistent with the simulation predictions, the data showed that the synthesis of Hla succeeded the synthesis of protein A and was strongly induced as soon as the yield of RNAIII became sufficiently high after 5 h of growth with a slight delay of 1 h (Figure 5A), as previously described (41–43). We also observed the same profile for the secreted form of Hla (Supplementary Figure S2A). These data are well correlated with the *hla* mRNA expression pattern (Supplementary Figure S1). For *rot* mRNA, its steady state level was almost identical in the wild-type and mutant strains (Supplementary Figure S1), consistent with the fact that RNAIII-dependent repression of *rot* occurs primarily at the translational level (27–28,39–40,44). Deletion of RNAIII significantly enhanced the synthesis of Rot (Supplementary Figure S2B). However, Rot synthesis decreased at the stationary phase of growth, probably owing to the effect of its negative autoregulation (27,40). Altogether, the repression of protein A synthesis in RN6390 coincide with RNAIII induction and with the decrease of Rot synthesis after 4 h of growth, and the activation of Hla is achieved after a delay of 1 h. This coordinated temporal regulation was lost in the two mutant strains (Figure 5B and C). Deletion of *rot* caused a strong repression of protein A at the beginning of growth while hla synthesis was significantly enhanced after 4 h of growth. The production of Hla was observed earlier in the Δrot mutant strain than in RN6390 (Figure 5B), consistent with the prediction of the model (Figure 4). The deletion of RNAIII in the other mutant induced a constitutive expression of protein A while Hla synthesis was inhibited. All in all, these data showed the importance of both Rot and RNAIII for the coordinated expression of *spa* and *hla* during growth.

#### Filtering of transient signals

**Figure 6.**
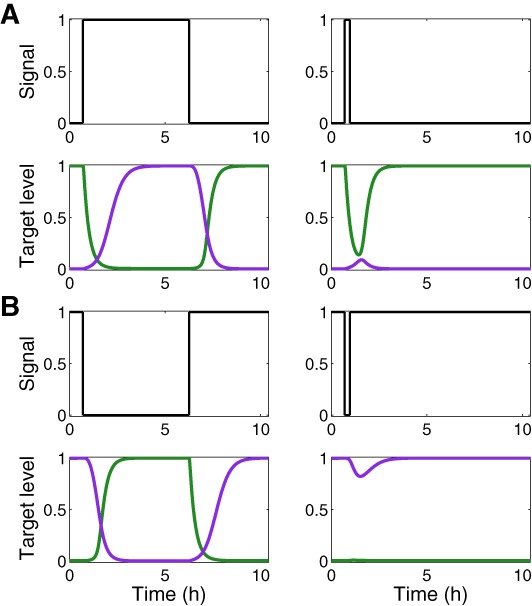
Filtering of transient signals by the DSS. Shown are normalized expression levels of target 1 (green) and target 2 (purple) in a DSS, following prolonged and transient ON signals (**A**) and OFF signals (**B**), obtained from simulations based on the deterministic model. The delayed target dynamics of the DSS leads to the filtering of transient ON signals by target 2 and transient OFF signals by target 1. The parameter values used in these simulations are reported in Table S1.

In many biological contexts, an efficient regulatory circuit is one that does not respond to transient changes in the external signal but only to persistent signals. To characterize the DSS in this respect, we ran the simulations when the signal was invoked for short and long periods, and followed the protein expression pattern of target genes 1 and 2. Only persistent signals yielded the coordinated change in the expression of proteins encoded by genes 1 and 2, for both the ON (Figure 6A) and OFF (Figure 6B) steps.

#### Prevention of expression leakage

**Figure 7.**
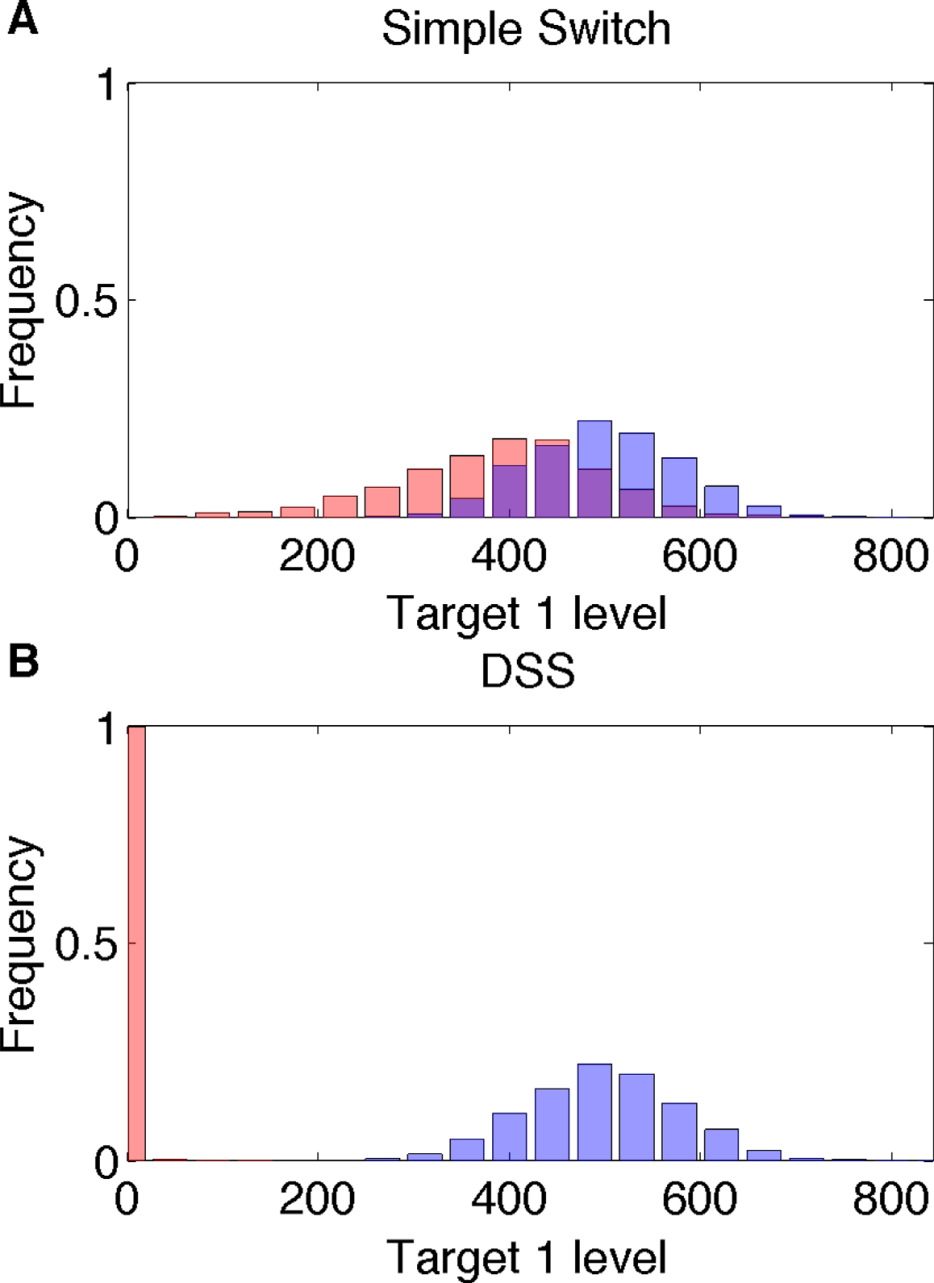
Distribution of target 1 protein level. Shown are the distribution of the protein levels (number of moecules) of target 1 upon ON step (pink bars) and OFF step (blue bars) for (**A**) a Simple TF Switch (a DSS structure without sRNA–targets interactions) and (**B**) a DSS, obtained from stochastic simulations. During OFF step, the DSS and the Simple Switch are functionally identical. During ON step, under DSS regulation, stochastic variations in target 1 level are substantially reduced (compared to DSS OFF step and compared to Simple Switch ON step), since the target is regulated at both transcriptional and post-transcriptional levels. The parameter values used in these simulations are reported in Table S1.

Transcription and translation are noisy processes and are susceptible to leakage, implying a low level of gene expression even when regulatory repression is in play. Hence, the effectiveness of a regulatory circuit can be evaluated by its ability to prevent leaky gene expression. A post-transcriptional regulation level, working coherently with a transcriptional regulation level, is expected to reduce the expression leakage of both targets due to the fact that deactivation of the target genes is enforced by two layers of regulation (45), compared to target regulation by a Simple Switch ( see Supplementary Material). Leakage reduction by the double layered regulation can be readily observed in Figure 2C, where during ON step, the leaking mRNAs of target 1 are sequestered by the top sRNA regulator, decreasing the protein level of target 1 (Figure 2B). In order to show this quantitatively, we defined the leakage level of a gene as the ratio between the steady state level when it is shutdown and the maximal possible level (achieved with no regulation) of its protein. Using the model described in ‘Materials and Methods’ section, we derived a mathematical expression for the leakage levels of the target genes regulated by the DSS. From this, we could directly infer that the leakage level of genes regulated by the DSS is indeed lower than the leakage levels of genes regulated by only one of the two regulatory layers (either the sRNA or the TF, see ‘Materials and Methods’ section and Supplementary Material). Thus, the combination of transcriptional and post-transcriptional layers of regulation reduces the leakage level of both targets, compared to single layered regulation. Using stochastic analysis (‘Materials and Methods’ section), it is evident that in addition to a decrease in leakage of target 1 during ON step, the variation in its level, compared to a Simple sRNA Switch, reduces as well (Figure 7).

**Figure 8.**
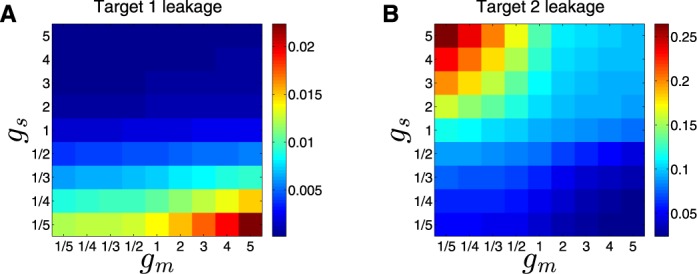
Prevention of expression leakage by the DSS. Shown are leakage levels (represented by a color scale) of target 1 (**A**) and 2 (**B**), as a function of fold-changes in RNA generation rates of the top sRNA regulator (*g_S_*, y-axis) and the bottom TF regulator (*g_m_, x*-axis), obtained from simulations based on the deterministic model. All other parameter values are kept constant, so that the expression level of the regulators, and hence their strength, depends on the change in their RNA generation rates. Sustaining low expression leakage for both targets requires a fine balance between the relative strengths of the two regulators involved in the DSS. The parameter values used in these simulations are reported in Table S1.

We next examined how the balance between the strengths of the two regulation layers (transcriptional and post-transcriptional) affects the leakage of the DSS targets. The strength of a regulation layer is determined by the level of the respective regulators, the sRNA and TF, which depends on their generation and degradation rates, and their binding to their targets. For simplicity, we kept all parameter values constant, and examined how the leakage of the targets is affected by changes in the RNA generation rates of the two regulators, as proxies for their strengths. The leakage level of target gene 1 (2) is a monotonically decreasing (increasing) function of the top regulator strength and is a monotonically increasing (decreasing) function of the bottom regulator strength. Indeed, as can be observed in Figure 8, a DSS with a strong (weak) top regulator and a weak (strong) bottom regulator results in a low (high) expression leakage of gene 1 and high (low) expression leakage of gene 2. Consider, for example, the effect on gene 1 in a DSS in case of strong regulation by the sRNA and weak regulation by the TF. During ON step, gene 1 is heavily repressed by the strong top sRNA. In addition, the sRNA represses the activator TF, which is weak to begin with. Overall, gene 1 transcription and translation are strongly repressed, leading to a very low expression leakage. A DSS with two regulators of the same strength (either strong or weak) would result in intermediate expression leakage of both targets, as can be observed in Figure 8. We conclude that sustaining low expression leakage for both targets requires a fine balance between the relative strengths of the two regulators of a DSS.

### DSS variants in different cellular contexts

**Figure 9.**
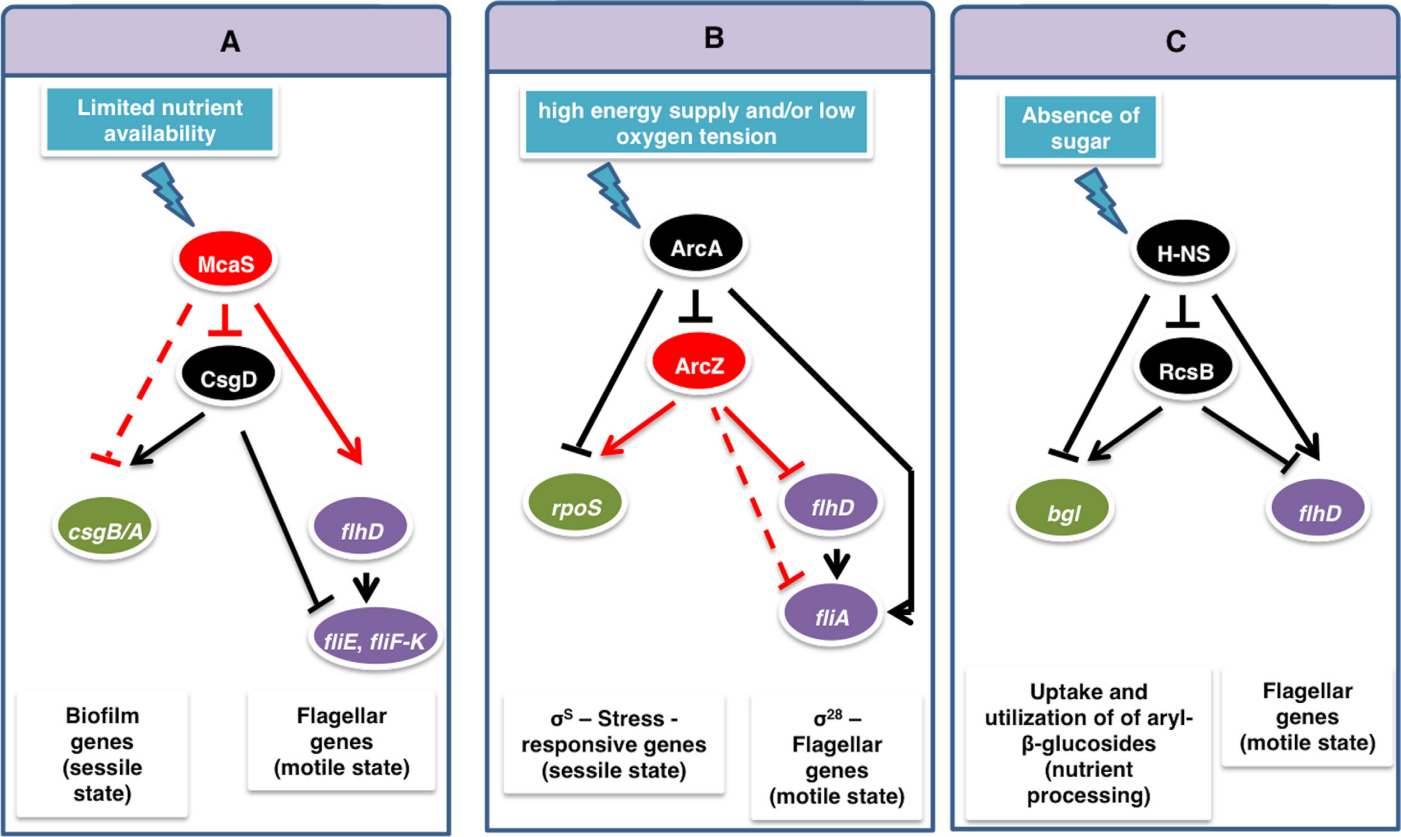
Variants of the DSS module in various cellular contexts in *Escherichia coli*. (**A**) A partial DSS variant defined by the top regulator sRNA McaS and the bottom regulator TF CsgD, controlling the switch between motile and sessile lifestyles. (**B**) A partial DSS variant defined by the TF ArcA as top regulator and the sRNA ArcZ as bottom regulator, controlling the switch between two sigma factors. (**C**) A DSS variant defined by the TFs H-NS and RcsB, controlling the switch between the uptake and utilization of existing nutrients and the active search for food when the local environment is nutrient-deprived. Arrows indicate positive regulation and T-shaped arrows indicate negative regulation (red for regulation by a sRNA and black for regulation by a TF). Solid and dashed lines represent experimentally verified and predicted interactions, respectively.

We can identify DSS structures automatically by searching such connected patterns in the cellular regulatory networks of organisms with sufficient number of reported regulatory interactions, such as *Escherichia coli*. Since a few *E. coli* sRNAs were shown to act also as activators, they open the door to possible identification of DSS structures. Our search has led us to discover several partial DSSs with sRNA as top regulator and TF as bottom regulator. One of these circuits is shown in Figure 9A and involves the sRNA McaS and the TF CsgD, playing a role in the switch between motile and sessile lifestyles (46–49). Taking a more general view of the DSS, there may be such circuits involving a top TF regulator and a bottom sRNA regulator. Indeed, we identified an intriguing DSS variant involving the top TF ArcA and the bottom sRNA ArcZ (Figure 9B), controlling the switch between *rpoS* and *fliA*, two genes encoding sigma factors that are active during different cellular states, where *fliA* is repressed under starvation or upon entry to stationary phase (50), and *rpoS* is activated under these conditions (51). The final example involves a complete transcriptional DSS, defined by the global regulator H-Ns as top regulator, the TF RcsB as bottom regulator, the *bgl* operon as target 1, and *flhD* as target 2 (Figure 9C). The *bgl* operon encodes all functions necessary for the regulated uptake and utilization of aryl-β-glucosides (52,53), while FlhD is the master regulator of flagellar genes. In the presence of sugar, RcsB activates *bgl* (54) and represses *flhD* (55). In the absence of sugar, Hns represses RcsB (56) and bgl (57) and activates flhD (58). This suggests that this transcriptional DSS controls the switch between the uptake and utilization of existing nutrients and the active search for food when the local environment is nutrient-deprived.

## DISCUSSION

In this study, we identified and analyzed an elaborate switching module termed the DSS (Figure 1) within the complex regulatory network of *S. aureus*, comprising a regulatory sRNA and a transcription factor, which governs the switch between gene expression programs leading to defensive and offensive phenotypes of the bacteria according to quorum sensing signal. We showed that the DSS exhibits special dynamical properties as a switching device (fine-tuned target expression coordination, tight regulation and filtering of transient signals) not exhibited by simpler switches that compose it (a DSS lacking either the TF regulator or the sRNA–targets interactions). The integration of transcriptional and post-transcriptional regulators, inversely activating one target and deactivating another target, guarantees a fine-tuned coordinated switch in the expression of these targets (targets 1 and 2), as we demonstrated both computationally (Figure 2) and experimentally under physiological conditions (Figure 5, Supplementary Figures S1 and S2). As we showed, the DSS guarantees that target 2 is up-regulated only following the down-regulation of target 1, resulting also in efficient filtering of transient signals (Figure 6). Consistent with the predictions of the model (Figures 2–4), we showed experimentally this fine-tuned gene expression coordination (Figure 5A), which is abolished when only one layer of regulation is active (Figure 5B), further substantiating the DSS special regulatory properties.

This fine-tuned coordination in gene expression stems from two independent mechanisms: the structural properties of the DSS and the stoichiometric nature of post-transcriptional regulation by sRNAs. The structure of the DSS comprises two combined multi-layered coherent FFLs, involving the shared transcriptional and post-transcriptional regulators of the two targets. Target 1 is controlled by a type II coherent multi-layered FFL. Upon the ON step the down-regulation of target 1 is accelerated by the top sRNA regulator, which binds to its mRNA for repression (45), while in the OFF step its up-regulation is delayed. Target 2 is controlled by a type IV coherent multi-layered FFL, creating a delay in its up-regulation upon ON step (59,60) and enhancement of its down-regulation upon the OFF step, due to the fast effect of decrease in the top sRNA regulator. The combination of enhancement of down-regulation of target 1 governed by the multi-layered FFL type II and delay in target 2 up-regulation governed by the multi-layered FFL type IV underlies the fine-tuned coordination in gene expression we observed both computationally and experimentally upon the ON step (Figures 2 and 5). The stoichiometric properties of post-transcriptional regulation by a sRNA provide an additional boost to the coordination between target 1 and target 2 protein expression upon the ON step, provided that the sRNA and target mRNA levels are comparable. The sRNA exerts its regulatory function by binding its mRNA targets, forming sRNA–mRNA complexes. As a result, as opposed to transcriptional regulation, the effectiveness of regulation depends not only on the level of the regulator but also on the level of its target mRNAs (45,61). This property is shown in Figure 2C, presenting the level of the sRNA–target mRNA complexes. Upon activation of the sRNA (ON step), the level of target 1 transcripts is maximal whereas the level of target 2 transcripts is minimal. Therefore, the sRNA mainly binds the prevalent mRNA molecules of target 1, leading to a fast decrease in the level of the encoded protein (following a fast increase in sRNA–mRNA complex level). As the level of target 1 mRNA decreases, more sRNA transcripts are available to bind the increasing number of mRNA molecules of target 2, which is now transcriptionally active due to the relief of the repression by the TF, enhancing the production of the protein product of target 2. Thus, upon ON step, the sRNA devotes most of its copies to only one of the targets, the one that is relevant at that stage of the switching process, implying that the stoichiometric nature of sRNA–mRNA interaction contributes to the observed coordination in the protein expression of targets controlled by the DSS. As we showed in Figure 3, changes in the values of different parameters, such as the sRNA generation rate, affect the time interval between the down regulation of target 1 and up-regulation of target 2, which can be potentially tuned by evolution to allow for optimal timing of the switching process. This is opposed to a Simple TF Switch (A DSS structure with no sRNA-targets interactions) and a Simple sRNA Switch (A DSS structure lacking a TF regulator), in which the changes in expression of the two targets are fixed to occur simultaneously (‘Materials and Methods’ section and Supplementary Material).

Pathogenic bacteria have evolved a variety of regulatory circuits that control the expression of virulence factors enabling them to colonize and survive during the host infection. In these networks, regulatory RNAs and transcriptional regulatory proteins generate intricate interactions (e.g. (19,62)). For instance, in *Vibrio cholerae*, four redundant Qrr sRNAs regulate the synthesis of two master quorum-sensing regulatory proteins, which operate either at low cell density (AphA) or at high cell density (HapR). This interplay between the transcriptional regulatory proteins and Qrr sRNAs generates a reciprocal gradient of AphA and HapR expression to establish the quorum sensing gene expression patterns at low and high density levels (63,64). Another characteristic example is the regulatory module involving the master post-transcriptional regulatory proteins CsrA/RsmA, which regulates primary and secondary metabolic pathways, biofilm formation, motility, virulence of pathogens, quorum sensing and stress response systems. CsrA binds to conserved and repeated AGG sequences in its target mRNAs to alter their translation and/or turnover. In γ-proteobacteria, several sRNAs sequester multiple CsrA/RsmA away from mRNA targets. Interestingly, the Csr system is based on extensive autoregulatory and feedback loops and the interaction of the Csr system with transcriptional regulatory networks was shown to result in a variety of cellular responses (65). The DSS we have identified in *S. aureus*, involving RNAIII and Rot, controls the effective transition of the pathogen from a defensive mode (chronic infection mode associated with biofilm formation) to an offensive mode (acute infection) following quorum sensing signal (35). Here we demonstrated the functionality of this switch in *S. aureus* under physiological conditions.

The experiments have been performed on RN6390 strain, a laboratory strain that is Sigma B-deficient (35) and a strong producer of RNAIII. As stated above, RNA-dependent regulation strongly relies on both the sRNA and target concentrations and therefore any variation in the expression/turnover rates of RNAIII or of its targets might affect the timing and the sequential synthesis of the virulence factors. For instance, in the strain SH1000/HG001 derived from the same parental strain (RN6390), but in which sigma B is restored, the expression of RNAIII was shown to be lowered and delayed, as is the repression of Protein A (target 1) and the induction of Hla synthesis (target 2) (e.g (66,67)). Interestingly, it has been described that the expression of RNAIII can vary up to 1000-fold among clinical isolates, primarily at the exponential growth phase (37,44,68). The low RNAIII producer strain UAMS-1 expresses factors that would favor biofilm formation and colonization (37). This supports the model of *agr* being important for full expression of virulence, notably during acute infection, while *agr* mutants would be positively selected in chronic infections and dormant states (69). Furthermore, the *agr* locus has diverged among *S. aureus* strains leading to four distinct groups, and specific differences in *agr* autoinduction in the transcriptional regulator Rot and virulence gene regulation have been recently observed (40). Several studies have also shown that it is difficult to predict the expression of RNAIII *in vivo* but the vast majority of clinical isolates from acute infections express RNAIII (68,70–72). These isolate-specific variations in RNAIII levels between the various isolates might affect the leakage level and the time interval between the repression of the defensive genes and activation of the offensive genes. In addition, other global regulators such as SarA (staphylococcal accessory regulator), the two-component system saeRS and the stress-response SigB (Sigma factor B) also contributed to this regulatory switch independently of the quorum sensing signal. These multiple regulators might provide advantages to the cell population in order to produce the virulence factors in response to multiple signals (11,73).

Our findings have implications to synthetic biology, suggesting that the current module toolbox, which thus far mainly contained simple modules such as toggle switches and FFLs (74,75), may be extended by combinations of such modules. In this context, the DSS may be used as a template for an effective switching device, designed to control well-coordinated up- and down-regulation of specific genes in response to an external signal. Indeed, we found similar regulatory structures in *E. coli*, controlling key decision-making processes, such as the transition between motile and sessile (46) or aerobic and anaerobic (76) lifestyles, or the transition between the uptake and utilization of existing nutrients and the active search for food when the local environment is nutrient-deprived (56,77) (Figure 9). Further comparative topological and dynamical analyses of regulatory networks in various pathogenic bacteria are expected to enlighten the common and diverged mechanisms involved in host–pathogen interactions.

## SUPPLEMENTARY DATA

Supplementary data are available at NAR Online.

SUPPLEMENTARY DATA
