# Supplementary material for: A defense-offense multi-layered regulatory switch in a pathogenic bacterium
Source: Nucleic Acids Res. 2015 Jan 27;43(3):1357–69. doi: 10.1093/nar/gkv001 (PMC4330369; doi:10.1093/nar/gkv001)
Supplement: SUPPLEMENTARY DATA [file supp_43_3_1357__index.html]

A defense-offense multi-layered regulatory switch in a pathogenic bacterium — SUPPLEMENTARY DATA 

# A defense-offense multi-layered regulatory switch in a pathogenic bacterium

## SUPPLEMENTARY DATA

**Files in this Data Supplement:**

- SUPPLEMENTARY DATA
