# Supplementary material for: A defense-offense multi-layered regulatory switch in a pathogenic bacterium
Source: Nucleic Acids Res. 2015 Jan 27;43(3):1357–69. doi: 10.1093/nar/gkv001 (PMC4330369; doi:10.1093/nar/gkv001)
Supplement: SUPPLEMENTARY DATA [file supp_gkv001_nar-02318-n-2014-File011.pdf]

A Defense-offense multi-layered regulatory switch in a pathogenic bacterium - Supplementary Material

Mor Nitzan[1,2], Pierre Fechter[3], Asaf Peer[2], Yael Altuvia[2], Delphine Bronesky[3], François Vandenesch[4], Pascale Romby[3], Ofer Biham[1], Hanah Margalit[2,*]

1. Racah Institute of Physics, The Hebrew University, Jerusalem 91904, Israel.
2. Department of Microbiology and Molecular Genetics, IMRIC, Faculty of Medicine, The Hebrew University, Jerusalem 91120, Israel.
3. Architecture et Réactivité de l'ARN, Université de Strasbourg, CNRS, IBMC, Strasbourg F-67084, France.
4. CIRI, International Center for Infectiology Research; Inserm, U1111; École Normale Supérieure de Lyon; Université Lyon 1; CNRS, UMR5308; Lyon, France.

* Corresponding Author: Hanah Margalit
e-mail: hanahm@ekmd.huji.ac.il
Tel.: 972-2-6758614
Fax: 972-2-6757308.

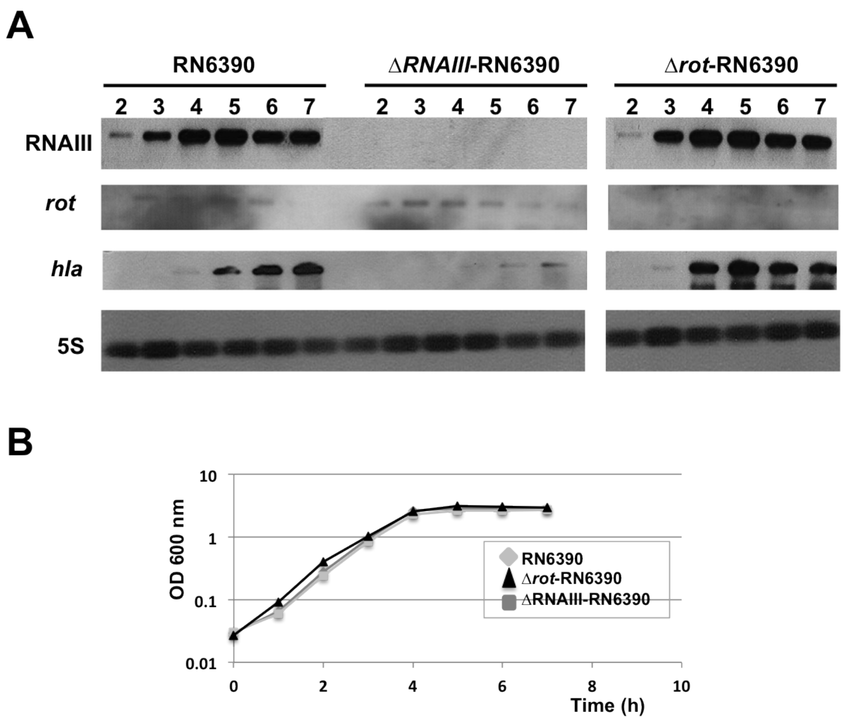

Figure S1: **Dynamics of DSS components determined experimentally. (A)** Northern analysis showing the levels of RNAIII, and of the mRNAs encoding the transcriptional repressor of toxins Rot (*rot*) and α-hemolysin (*hla*) in RN6390, and the mutant (Δrot-RN6390, ΔrnaIII-RN6390) strains. Total RNAs were extracted hourly (h) at different times of growth. 5S rRNA was used as an internal loading control. The same crude extracts were run on various gels to perform the Northern blots. The experiments were reproduced at least three times from different samples with good reproducibility. **(B)** The growth curves were performed in TSB medium with RN6390 (♦) and the isogenic mutants ΔrnaIII-RN6390 (■) and Δrot-RN6390 (▲) strains.

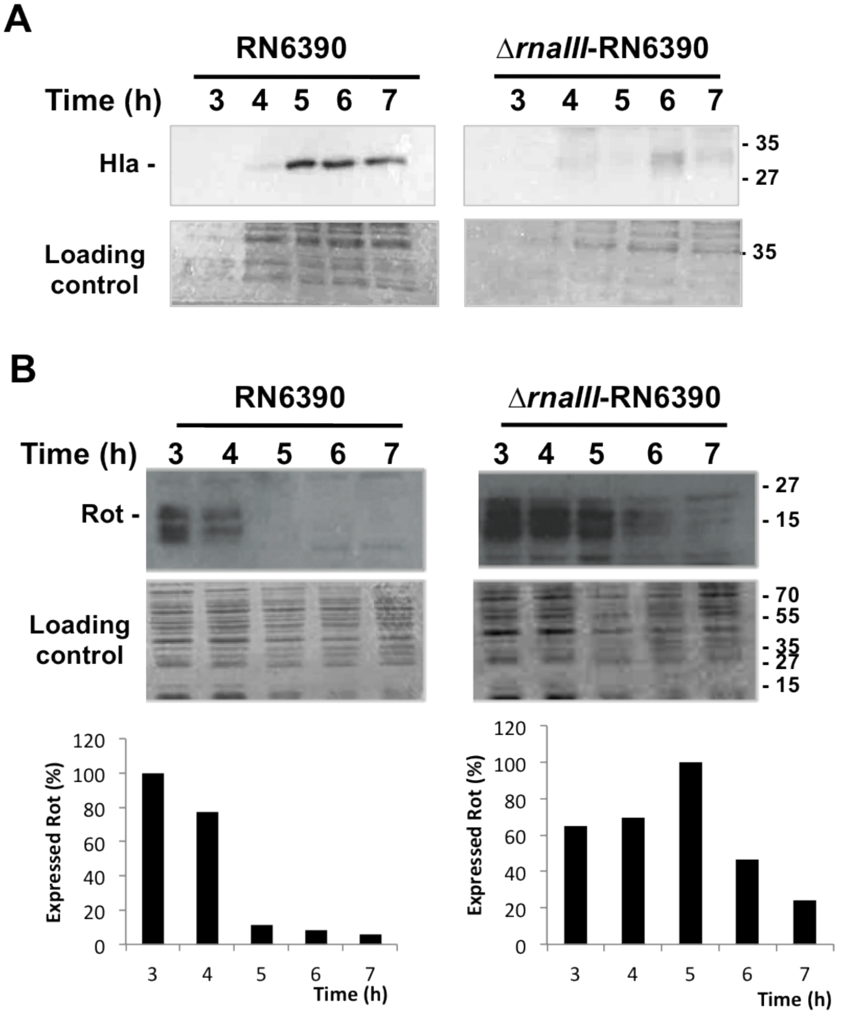

Figure S2: **Western blots of total as well as secreted protein extracts for hemolysin α (Hla) (A) and for the repressor of toxins Rot (B).** Protein extracts were prepared from the wild type strain RN6390 and the isogenic mutant strain deleted of *rnaIII* gene (ΔrnaIII-RN6390). The proteins were revealed by immunoblotting using polyclonal antibodies from either secreted protein extract **(A)** or from total protein cell extracts **(B)**. For the preparation of the secreted protein extracts, the same quantity of cells were used for centrifugation and identical volume of each supernatant was loaded on the gel electrophoresis for coomassie blue staining. The yields of the exoproteins in RN6390 vary with the growth curve because their synthesis is dependent on the quorum sensing system while their levels significantly decrease in the mutant ΔrnaIII-RN6390 strain as compared to RN6390. For the total protein extracts, the quantity of the proteins was controlled and adjusted in each lane, and verified by coomassie blue staining. The proteins were extracted at different times of growth. Molecular weight markers were run in parallel. The bands corresponding to Rot protein have been quantified using the software SAFA [1]. Several proteins from the loading controls, which do not vary during growth, have were used to normalize the data. Note that Rot protein is barely detected in RN6390 strain but the experiments were carried out three times with good reproducibility.

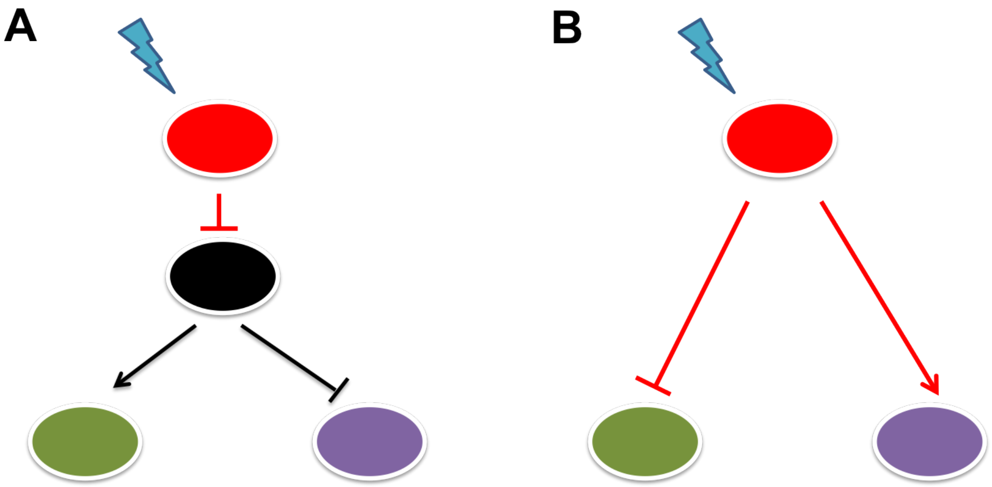

Figure S3: Simple switches. Structure and colours are analogous to a DSS structure (Figure 1 in the main text) for a Simple TF Switch (A DSS structure with no sRNA-targets interactions) **(A)** and a Simple sRNA Switch (A DSS structure with no TF regulator) **(B)**. The red circles indicate sRNAs and the black circle indicates a TF. Arrows indicate positive regulation and T-shaped arrows indicate negative regulation (red for regulation by a sRNA and black for regulation by a TF).

Process	Parameter symbol	Values used in simulations (s^{-1})	Reasoning / Reference
sRNA ON transcription rate	g_S	2 (range for Figure 8: 0.1-2.5, default 2)	*
sRNA OFF transcription rate	g_S	0	For simplicity, during OFF step the sRNA is assumed to not transcribe
TF transcription rate	g_m	0.03 (range for Figure 8: 0.006-0.15, default 0.03)	*
Targets transcription rate	g_m	0.03	*
mRNA translation rate	g_P	0.05	*
Free target 2 translation rate	$\tilde{g_P}$	0.005	We assumed that the translation rate of the mRNA with blocked RBS would be 0.1 of the rate of the unblocked mRNA
sRNA degradation rate	d_S	0.0008	*
mRNA degradation rate	d_m	0.003	*
Protein degradation rate	d_P	0.001	*
sRNA binding rate to target mRNAs	b_S	0.001	*
sRNA - mRNA complex degradation rate	d_{Sm}	0.003	We assumed the complex degradation rate to be comparable to the degradation rate of the mRNA
TF binding rate to target promoters	b_T	0.05	*
TF unbinding rate from target promoters	u_T	0.01	*

Table S1: Rate constants of the mathematical model and the values used in the simulations. Default values (*) are within the range reported in [2].

	**Relevant characteristics**	**References**
***S. aureus* strains**		
RN6390	Derivative of 8325-4, rsbU deficient, *agr* positive	[3]
LUG950	- RN6390 : ΔrnaIII region::kanamycin resistance gene	[4]
**Oligonucleotides for Northern**		
T7-Hla for	GATCCCGCAAATTAATACGACTCAC-TATAGGGTTTGAGCTACTTGAT-TATCAGGTAGTTGCA	
Hla-rev	CATATTAATGAATGAATCCGTCGCTAATGCCGC	
T7-Spa for	ATCCCGCAAATTAATACGACTCACTATAGGGCGT-TACGTTGCGCTTCGTTAAGTTAGGCA	
Spa rev	TTAGGTACATTACTTATATCTGGTGGCGTA	
T7-*rot*for	GATCCCGCAAATTAATACGACTCAC-TATAGGGCTTGATTCATCCTGATTTTTTTGGTG-CAAAGTC	
Rot rev	CTGCATTAACTGTATTTCCGGCAACTTCTTATGC	
T7-RNAIII for	GATCCCGCAAATTAATACGACTCAC-TATAGGGCACTGAGTCCAAGGAAACTAACTCTAC-TAGC	
RNAIII rev	CACAGAGATGTGATGGAAAATAGTTGATGAGTTG	

Table S2: Strains and oligonucleotides used in this study

The dynamics of Simple Switches

Deterministic models

Similarly to the deterministic model described in the main text for the DSS, the rate equations describing a Simple TF Switch (a DSS structure missing its sRNA-target 1 and sRNA-target 2 interactions, as shown in Figure S3A) take the form:

$$\frac{dN_S}{dt} = g_s - b_S N_S N_{mT} - d_S N_S \tag{S1a}$$

$$\frac{dN_{mT}}{dt} = g_m - b_S N_S N_{mT} - d_m N_{mT} \tag{S1b}$$

$$\frac{dN_{ST}}{dt} = b_S N_S N_{mT} - d_{Sm} N_{ST} \tag{S1c}$$

$$\frac{dN_{PT}}{dt} = g_P N_{mT} - d_P N_{PT} - [b_T N_{PT}(1 - N_{T1}) - u_T N_{T1}] - [b_T N_{PT}(1 - N_{T2}) - u_T N_{T2}] \tag{S1d}$$

$$\frac{dN_{Ti}}{dt} = b_T N_{PT}(1 - N_{Ti}) - u_T N_{Ti}, \quad i = 1, 2 \tag{S1e}$$

$$\frac{dN_{m1}}{dt} = g_m N_{T1} - d_m N_{m1} \tag{S1f}$$

$$\frac{dN_{m2}}{dt} = g_m(1 - N_{T2}) - d_m N_{m2} \tag{S1g}$$

$$\frac{dN_{Pi}}{dt} = g_P N_{mi} - d_P N_{Pi}, \quad i = 1, 2 \ , \tag{S1h}$$

The rate equations describing a Simple sRNA Switch (a DSS structure lacking the transcription factor regulator, as shown in Figure S3B) take the form:

$$\frac{dN_S}{dt} = g_s - b_S N_S (N_{m1} + N_{m2}) - d_S N_S \tag{S2a}$$

$$\frac{dN_{m1}}{dt} = g_{m0} - b_S N_S N_{m1} - d_m N_{m1} \tag{S2b}$$

$$\frac{dN_{m2}}{dt} = g_m - b_S N_S N_{m2} - d_m N_{m2} \tag{S2c}$$

$$\frac{dN_{Si}}{dt} = b_S N_S N_{mi} - d_{Sm} N_{Si}, \quad i = 1, 2 \tag{S2d}$$

$$\frac{dN_{P1}}{dt} = g_P N_{m1} - d_P N_{P1} \tag{S2e}$$

$$\frac{dN_{P2}}{dt} = g_P N_{S2} - d_P N_{P2} \ . \tag{S2f}$$

where g_{m0} is the target basal transcription rate. In the simulation results presented below, $g_{m0} = g_m/10$.

Stochastic models

Analogously to the deterministic analysis, the state of the Simple TF Switch can be described by the state vector $(N_S, N_{mT}, N_{ST}, N_{PT}, N_{T1}, N_{T2}, N_{m1}, N_{m2}, N_{P1}, N_{P2})$. The master equation corresponding to the mathematical model of the Simple TF Switch, presented above (Eq. (S1)), describing the time dependence of the probability distribution $P(N_S, N_{mT}, N_{ST}, N_{PT}, N_{T1}, N_{T2}, N_{m1}, N_{m2}, N_{P1}, N_{P2})$, takes the form:

$$
\begin{aligned}
&\frac{\partial}{\partial t}P(N_S, N_{mT}, N_{ST}, N_{PT}, N_{T1}, N_{T2}, N_{m1}, N_{m2}, N_{P1}, N_{P2}) = \\
&\quad b_S(N_S+1)(N_{mT}+1)P(N_S+1, N_{mT}+1, N_{ST}-1, \cdot, \cdot, \cdot, \cdot, \cdot, \cdot, \cdot) - b_S N_S N_{mT} P(\cdot, \cdot, \cdot, \cdot, \cdot, \cdot, \cdot, \cdot, \cdot, \cdot) \\
&\quad + b_T(N_{PT}+1)\delta_{N_{T1},1}P(\cdot, \cdot, \cdot, N_{PT}+1, 0, \cdot, \cdot, \cdot, \cdot, \cdot) + b_T(N_{PT}+1)\delta_{N_{T2},1}P(\cdot, \cdot, \cdot, N_{PT}+1, \cdot, 0, \cdot, \cdot, \cdot, \cdot) \\
&\quad - b_T N_{PT}\delta_{N_{T1},0}P(\cdot, \cdot, \cdot, \cdot, 0, \cdot, \cdot, \cdot, \cdot, \cdot) - b_T N_{PT}\delta_{N_{T2},0}P(\cdot, \cdot, \cdot, \cdot, \cdot, 0, \cdot, \cdot, \cdot, \cdot) \\
&\quad - u_T\delta_{N_{T1},1}P(\cdot, \cdot, \cdot, \cdot, 1, \cdot, \cdot, \cdot, \cdot, \cdot) - u_T\delta_{N_{T2},1}P(\cdot, \cdot, \cdot, \cdot, \cdot, 1, \cdot, \cdot, \cdot, \cdot) \\
&\quad + u_T\delta_{N_{T1},0}P(\cdot, \cdot, \cdot, N_{PT}-1, 1, \cdot, \cdot, \cdot, \cdot, \cdot) + u_T\delta_{N_{T2},0}P(\cdot, \cdot, \cdot, N_{PT}-1, \cdot, 1, \cdot, \cdot, \cdot, \cdot) \\
&\quad + g_S P(N_S-1, \cdot, \cdot, \cdot, \cdot, \cdot, \cdot, \cdot, \cdot, \cdot) + g_m P(\cdot, N_{mT}-1, \cdot, \cdot, \cdot, \cdot, \cdot, \cdot, \cdot, \cdot) \\
&\quad + g_m\delta_{N_{T1},1}P(\cdot, \cdot, \cdot, \cdot, \cdot, \cdot, N_{m1}-1, \cdot, \cdot, \cdot) + g_m\delta_{N_{T2},0}P(\cdot, \cdot, \cdot, \cdot, \cdot, \cdot, \cdot, N_{m2}-1, \cdot, \cdot) \\
&\quad + g_P N_{mT} P(\cdot, \cdot, \cdot, N_{PT}-1, \cdot, \cdot, \cdot, \cdot, \cdot, \cdot) \\
&\quad + g_P N_{m1} P(\cdot, \cdot, \cdot, \cdot, \cdot, \cdot, \cdot, \cdot, N_{P1}-1, \cdot) + g_P N_{m2} P(\cdot, \cdot, \cdot, \cdot, \cdot, \cdot, \cdot, \cdot, \cdot, N_{P2}-1) \\
&\quad - [g_S + g_m + g_P N_{mT} + g_m\delta_{N_{T1},1} + g_m\delta_{N_{T2},0} + g_P N_{m1} + g_P N_{m2}]P(\cdot, \cdot, \cdot, \cdot, \cdot, \cdot, \cdot, \cdot, \cdot, \cdot) \\
&\quad + d_S(N_S+1)P(N_S+1, \cdot, \cdot, \cdot, \cdot, \cdot, \cdot, \cdot, \cdot, \cdot) + d_m(N_{mT}+1)P(\cdot, N_{mT}+1, \cdot, \cdot, \cdot, \cdot, \cdot, \cdot, \cdot, \cdot,) \\
&\quad + d_P(N_{PT}+1)P(\cdot, \cdot, \cdot, N_{PT}+1, \cdot, \cdot, \cdot, \cdot, \cdot, \cdot) \\
&\quad + d_m(N_{m1}+1)P(\cdot, \cdot, \cdot, \cdot, \cdot, \cdot, N_{m1}+1, \cdot, \cdot, \cdot) + d_m(N_{m2}+1)P(\cdot, \cdot, \cdot, \cdot, \cdot, \cdot, \cdot, N_{m2}+1, \cdot, \cdot) \\
&\quad + d_P(N_{P1}+1)P(\cdot, \cdot, \cdot, \cdot, \cdot, \cdot, \cdot, \cdot, \cdot, \cdot, N_{P1}+1, \cdot) + d_P(N_{P2}+1)P(\cdot, \cdot, \cdot, \cdot, \cdot, \cdot, \cdot, \cdot, \cdot, \cdot, \cdot, N_{P2}+1) \\
&\quad - [d_S N_S + d_m N_{mT} + d_P N_{PT} + d_m N_{m1} + d_m N_{m2} + d_P N_{P2} + d_P N_{P1}]P(\cdot, \cdot, \cdot, \cdot, \cdot, \cdot, \cdot, \cdot, \cdot, \cdot, \cdot) \ .
\end{aligned} \tag{S3}
$$

The state of the Simple sRNA Switch can be described by the state vector $(N_S, N_{m1}, N_{m2}, N_{S1}, N_{S2}, N_{P1}, N_{P2})$. The master equation corresponding to the mathematical model presented above (Eq. (S2)) takes the form:

$$
\begin{aligned}
&\frac{\partial}{\partial t}P(N_S, N_{m1}, N_{m2}, N_{S1}, N_{S2}, N_{P1}, N_{P2}) = \\
&\quad + b_S(N_S+1)(N_{m1}+1)P(N_S+1, N_{m1}+1, \cdot, N_{S1}-1, \cdot, \cdot, \cdot) \\
&\quad + b_S(N_S+1)(N_{m2}+1)P(N_S+1, \cdot, N_{m2}+1, \cdot, N_{S2}-1, \cdot, \cdot) \\
&\quad - b_S N_S[N_{m1}+N_{m2}]P(\cdot, \cdot, \cdot, \cdot, \cdot, \cdot, \cdot) + g_S P(N_S-1, \cdot, \cdot, \cdot, \cdot, \cdot, \cdot) \\
&\quad + g_{m0}P(\cdot, N_{m1}-1, \cdot, \cdot, \cdot, \cdot, \cdot) + (g_{m0}+g_m)P(\cdot, \cdot, N_{m2}-1, \cdot, \cdot, \cdot, \cdot) \\
&\quad + g_P N_{m1}P(\cdot, \cdot, \cdot, \cdot, \cdot, N_{P1}-1, \cdot) + g_P N_{S2}P(\cdot, \cdot, \cdot, \cdot, \cdot, \cdot, N_{P2}-1) \\
&\quad - [g_S + (g_{m0}+g_m) + g_{m0} + g_P N_{m1} + g_P N_{S2}]P(\cdot, \cdot, \cdot, \cdot, \cdot, \cdot, \cdot) \\
&\quad + d_S(N_S+1)P(N_S+1, \cdot, \cdot, \cdot, \cdot, \cdot, \cdot) \\
&\quad + d_m(N_{m1}+1)P(\cdot, \cdot, \cdot, \cdot, \cdot, \cdot, \cdot) + d_m(N_{m2}+1)P(\cdot, \cdot, N_{m2}+1, \cdot, \cdot, \cdot, \cdot) \\
&\quad + d_{Sm}(N_{S1}+1)P(\cdot, \cdot, \cdot, N_{S1}+1, \cdot, \cdot, \cdot) + d_{Sm}(N_{S2}+1)P(\cdot, \cdot, \cdot, \cdot, N_{S2}+1, \cdot, \cdot) \\
&\quad + d_P(N_{P1}+1)P(\cdot, \cdot, \cdot, \cdot, \cdot, N_{P1}+1, \cdot) + d_P(N_{P2}+1)P(\cdot, \cdot, \cdot, \cdot, \cdot, \cdot, N_{P2}+1) \\
&\quad - [d_S N_S + d_m N_{m1} + d_P N_{P2} + d_P N_{P1} + d_{Sm}N_{S1} + d_{Sm}N_{S2} + d_m N_{m2}]P(\cdot, \cdot, \cdot, \cdot, \cdot, \cdot, \cdot) \ ,
\end{aligned} \tag{S4}
$$

where, for convenience, if there is no change in the state of the variables within the distribution, they are marked by a dot ('·').

We used the Gillespie algorithm [5], as in the main text, to generates 'paths' of the stochastic process.

Comparing a DSS and Simple Switches

Here we will discuss the differences between a DSS and Simple Switches in terms of leakage, target coordination, and response to transient signals.

Leakage

The leakage level of a target is defined as the ratio between the steady state level under conditions in which the target is repressed and maximal possible level (achieved with no negative regulation) of its protein, $L_X = \frac{N_X}{{N_X}^{max}}$.

For a Simple TF Switch, the leakage of target gene 1 (when the sRNA is activated) is given by:

$$L_1 = \frac{N_{P1}}{{N_{P1}}^{max}} = \frac{N_{P1}}{\frac{g_m}{d_m}\frac{g_P}{d_P}} = \frac{\frac{b_T}{u_T}N_{PT}}{1+\frac{b_T}{u_T}N_{PT}} \ . \tag{S5}$$

Similarly, the leakage of target gene 2, when the sRNA is not activated, is given by

$$L_2 = \frac{N_{P2}}{{N_{P2}}^{max}} = \frac{N_{P2}}{\frac{g_m}{d_m}\frac{g_P}{d_P}} = \frac{1}{1+\frac{b_T}{u_T}N_{PT}} \ . \tag{S6}$$

For a Simple sRNA Switch, the leakage of target gene 1 is given by:

$$L_1 = \frac{N_{P1}}{{N_{P1}}^{max}} = \frac{N_{P1}}{\frac{g_m}{d_m}\frac{g_P}{d_P}} = \frac{g_{m0}}{g_m}\frac{1}{1+\frac{b_s}{d_m}N_S} \ . \tag{S7}$$

The leakage of target gene 2 is given by:

$$L_2 = \frac{N_{P2}}{{N_{P2}}^{max}} = \frac{N_{P2}}{\frac{g_m}{d_m}\frac{g_P}{d_P}} = \frac{b_S N_S}{d_{Sm}(1+\frac{b_S}{d_m}N_S)} \ . \tag{S8}$$

Therefore, in general, the leakage levels of Simple Switches are higher than that of the leakage of a DSS, as appears in Materials and Methods Section in the main text.

Target coordination

The rate equation describing the total target mRNA level in a Simple TF Switch, according to Eq. S1:

$$\frac{d(N_{m1}+N_{m2})}{dt} = g_m - d_m(N_{m1}+N_{m2}) \ . \tag{S9}$$

The rate equation describing the total (functional) target mRNA level in a Simple sRNA Switch, in case $d_{Sm} = d_m$, according to Eq. S2:

$$\frac{d(N_{m1}+N_{S2})}{dt} = g_{m0} - d_m(N_{m1}+N_{S2}) \ . \tag{S10}$$

Total (functional) target mRNA level is thus independent of the regulators' level for both Simple Switches. Therefore, the two targets in the Simple Switches are inherently symmetrical in their individual incline and decline upon changes in external signal. This is opposed to the case of a DSS, as presented in the Materials and Methods Section in the main text.

References

[1] A Laederach, R Das, Q Vicens, SM Pearlman, M Brenowitz, D Herschlag, and RB Altman. Semiautomated and rapid quantification of nucleic acid footprinting and structure mapping experiments. *Nature protocols*, 3(9):1395–1401, 2008.

[2] M Nitzan, Y Shimoni, O Rosolio, H Margalit, and O Biham. Stochastic analysis of bistability in coherent mixed feedback loops combining transcriptional and post-transcriptional regulations. *arXiv:1412.0986 q-bio.MN*, 2014.

[3] HL Peng, RP Novick, B Kreiswirth, J Kornblum, and PM Schlievert. Cloning, characterization, and sequencing of an accessory gene regulator (*agr*) in *Staphylococcus aureus*. *Journal of Bacteriology*, 170(9):4365–4372, 1988.

[4] J Gagnaire, O Dauwalder, S Boisset, D Khau, A Freydière, F Ader, M Bes, G Lina, A Tristan, ME Reverdy, A Marchand, T Geissmann, Y Benito, G Durand, JP Charrier, J Etienne, M Welker, A Van Belkum, and F Vandenesch. Detection of *Staphylococcus aureus* delta-toxin production by whole-cell maldi-tof mass spectrometry. *PloS one*, 7(7):e40660, 2012.

[5] DT Gillespie. Exact stochastic simulation of coupled chemical reactions. *The journal of physical chemistry*, 81(25):2340–2361, 1977.
